# Multidisciplinary treatment of follicular thyroid carcinoma with hepatic and pulmonary metastases: a case report and literature review

**DOI:** 10.3389/fmed.2026.1848111

**Published:** 2026-06-05

**Authors:** Zhongyao Wang, Tian Wen, Zixin Li, Jingdong Li

**Affiliations:** 1 Department of Hepatobiliary Surgery, Affiliated Hospital of North Sichuan Medical College, Nanchong, Sichuan, China; 2 Department of Oncology, Affiliated Hospital of North Sichuan Medical College, Nanchong, Sichuan, China

**Keywords:** FT-UMP, liver metastasis, lung metastasis, MDT, follicular thyroid carcinoma

## Abstract

**Purpose:**

To determine the clinical diagnostic features, molecular basis, and comprehensive treatment strategies for a case of follicular thyroid carcinoma (FTC) with hepatic and pulmonary metastases that developed 8 years after surgery for a Follicular Tumor of Uncertain Malignant Potential (FT-UMP), and to provide a basis for long-term follow-up and individualized treatment of such borderline tumors.

**Background:**

FTC is the second most common subtype of differentiated thyroid cancer. Currently, the diagnosis and management of distant FTC metastases remain a critical challenge, particularly when they occur in rare locations, such as the liver.

**Case presentation:**

This article reports the case of a 56-year-old male patient. Within 8 years after the resection of the well-differentiated follicular neoplasm of FT-UMP, a single mass in the lateral lobe of the left liver and multiple nodules in both lungs were detected in the patient. Following a multidisciplinary team (MDT) discussion, the decision was made to perform a robot-assisted hepatectomy. Pathology confirmed the diagnosis of FTC metastasis. Subsequently, we formulated a comprehensive treatment plan including total thyroidectomy, radioactive iodine (RAI) therapy with ^131^I and endocrine treatment. Active surveillance was adopted for the lung metastases. Twelve months of follow-up showed no signs of recurrence.

**Conclusions:**

This case highlights that for patients with FT-UMP, continuous monitoring is also crucial for the early detection of metastasis. In complicated cases with liver and lung metastases from FTC, MDT collaboration is essential to formulate individualized treatment plans integrating multidisciplinary expertise and multimodal therapies.

## 1 Introduction

In recent years, the incidence of thyroid cancer has been gradually increasing, making it one of the most common malignant tumors. The second most common differentiated thyroid malignant tumor is FTC, which accounts for approximately 10% of all thyroid cancers. FTC may arise from FT-UMP, which carries an approximate 1% rate of progression to overt malignancy based on real-world data (1). The incidence of metastasis in patients with FTC ranges from 10% to 15%, with the lungs being the most common site of involvement, followed by the bones (2). Although it typically exhibits indolent behavior, the prognosis significantly worsens upon the occurrence of distant metastasis, with a 10 years post-metastasis survival rate of approximately 40% (3). Prognosis depends, to some extent, on the site and type of distant metastasis (4). It is relatively rare for it to metastasize to the brain and liver (5). Liver metastases are a rare occurrence in 0.5–1% of FTC (6). For patients with distant metastasis from thyroid cancer, treatment options include metastasectomy, endocrine therapy, ^131^I, targeted therapy, and radiotherapy. An individualized treatment plan is key. The 2025 ATA guidelines introduce the Diagnosis, risk/benefit Assessment, Treatment decisions, and response Assessment (DATA) iterative framework. Dynamic response assessment replaces static initial stratification. This provides a structured pathway for real-time decision-making in complex cases (7, 8). A total of 26 cases of FTC with liver metastasis have been reported in the literature. We review their diagnostic and therapeutic processes (Table 1).

**Table 1:** A review describes patients with FTC liver metastases.

Total cases treated	Other metastases	Liver metastasis (single/multiple)	Treatment of liver metastasis/es	Authors
1	Orbit	Multiple	Suppressive L-thyroxin and ^131^I	Babinska et al.(29)
6	NA	NA	All six cases received ^131^I, partial chemotherapy with tyrosine kinase inhibitors therapy, and 1 case underwent hepatectomy	Brient et al.(30)
1	No	Single	^131^I and hepatectomy	Djenic et al. (15)
1	NA	Single	Hepatectomy	Kondo et al. (31)
1	NA	Single	^131^I	Kraft (32)
1	NA	Single	Hepatectomy	Ligocka et al. (13)
1	No	Single	^131^I and hepatectomy	Ostrovsky et al. (33)
1	No	Single	^131^I	Battoo et al. (34)
1	No	Multiple	NA	Graves et al. (35)
1	No	Single	Hepatectomy	Kouso et al. (36)
1	Bone, breast	NA	NA	Tanriverdi et al. (37)
9	Bone in eight cases, lung in four cases	6 single, 3 multiple	^131^I in eight cases, five with radiotherapy, one with chemotherapy	Shah and Samuel (38)
1	Bone	Single	^131^I	Kelessis et al.(39)

NA, not analyzed.

## 2 Case report

### 2.1 Case presentation

**Figure 1:**
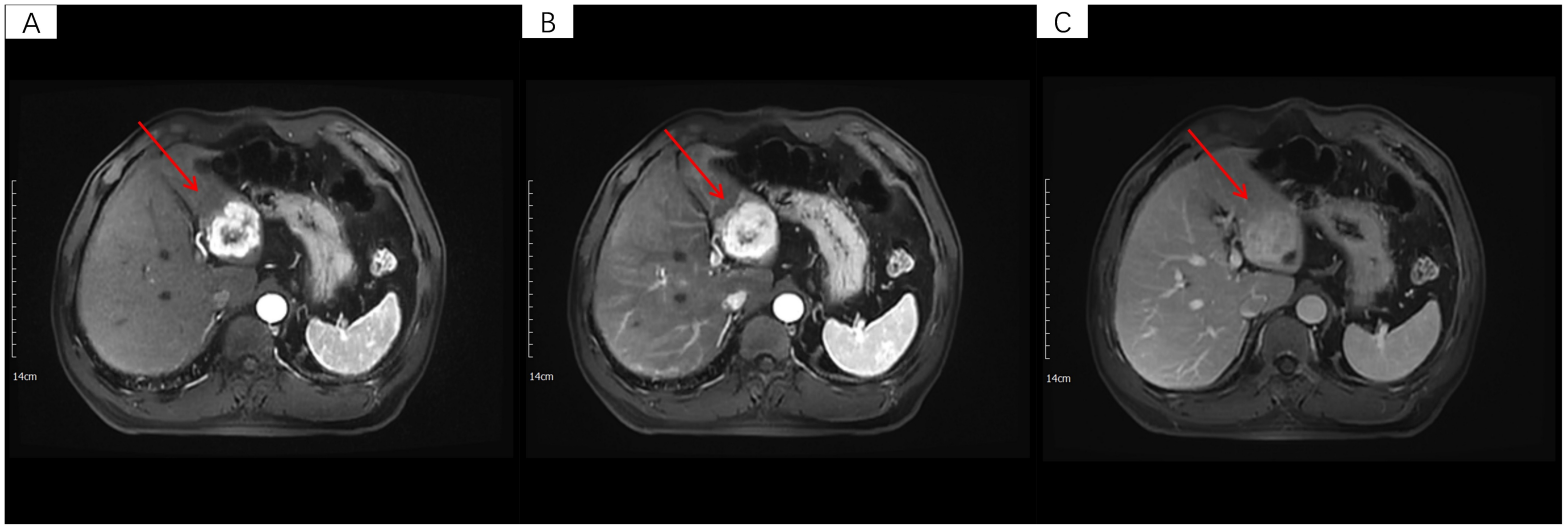
**(A)** early arterial phase, **(B)** late arterial phase, **(C)** delayed phase; the liver space-occupying lesion is located in the lateral segment of the left liver lobe (arrow).

A 56-year-old male patient was admitted in December 2024 due to a liver mass incidentally detected on a routine health examination. Ultrasonography had revealed a mass measuring approximately 4.0 cm × 4.5 cm in the left lateral lobe, with no signs of cirrhosis or fatty liver disease. He presented with no significant abdominal distension, pain, or skin jaundice. Abdominal examination revealed no positive signs. An upper abdominal MRI revealed a mass-like abnormal signal in the inferior segment of the left lateral liver lobe, measuring approximately 3.8 cm × 4.5 cm × 3.5 cm. On contrast-enhanced scans, the lesion showed marked heterogeneous enhancement in the arterial phase, with decreased enhancement in the portal venous and delayed phases (Figure 1). This mass was considered a neoplastic lesion. Serum tumor markers for liver malignancy were not significantly elevated. Thyroid function tests revealed a normal Thyroid-Stimulating Hormone (TSH) level. A chest CT indicated multiple nodules in both lungs, with the largest measuring approximately 8 mm × 6 mm.

Past medical history: The patient underwent left thyroid lobectomy and isthmusectomy 8 years ago for a solid nodule in the left thyroid lobe. Preoperative ultrasonography revealed a 2.2 cm × 1.9 cm heterogeneous hypoechoic nodule in the inferior pole of the left lobe, adjacent to the isthmus, with regular morphology, well-defined margins, and classified as TI-RADS 4a. Preoperative FNA suggested a Bethesda IV lesion. Intraoperative frozen section analysis indicated a follicular neoplasm. Postoperative pathology revealed cuboidal tumor cells with round nuclei, arranged in follicles of varying sizes. Focal nuclear crowding and nuclear grooving features were observed. No mitoses or necrosis were identified. The tumor had a complete fibrous capsule, with focal areas showing tumor contact without capsular penetration (all suspicious areas were confirmed by deeper sectioning). Immunohistochemically, the tumor cells were positive for TTF-1, thyroglobulin (Tg), CK7, CK19, and HBME-1, and negative for Galectin-3. CD31 staining outlined the vascular endothelium, with no evidence of intraluminal tumor invasion. These findings, together with the intact capsule and absence of capsular penetration, supported a diagnosis of FT-UMP.

**Figure 2:**
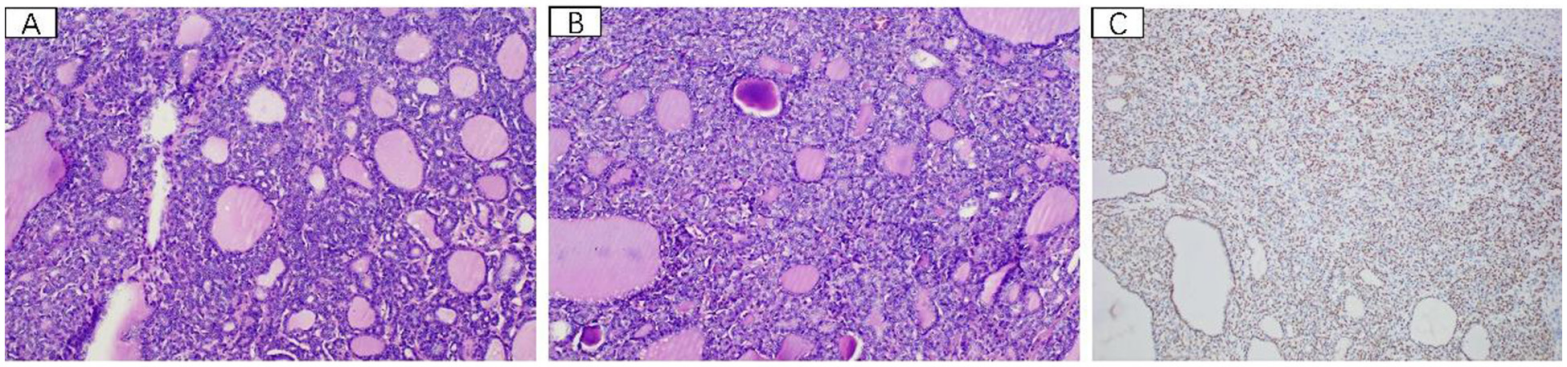
**(A, B)** Tumor cells in the liver formed follicular structures, some containing colloid (Hematoxylin and eosin, × 40). **(C)** Immunohistochemistry showed positivity for thyroglobulin ( × 40), which, in conjunction with the findings on hematoxylin and eosin staining, was consistent with metastatic follicular thyroid carcinoma. In addition, the tumor cells were positive for TTF-1, Tg, CK7, CK19, and CD56, and negativity for Galectin-3, with a Ki-67 proliferation index of approximately 5% in hotspot areas, further supporting this diagnosis.

After the initial assessment by the first MDT, the following decisions were made: (1) To perform a robot-assisted laparoscopic left lateral hepatectomy to clarify the nature of the liver mass. (2) To place the lung nodules under active surveillance for the time being. Postoperative pathological examination of the liver mass indicated metastatic thyroid carcinoma (Figure 2). Immunohistochemistry revealed positivity for TTF-1, Tg, CK7, CK19, and CD56, and negativity for Galectin-3, with a Ki-67 proliferation index of approximately 5% in hotspot areas. In the setting of distant metastasis, these findings support a diagnosis of metastatic FTC, as confirmed on review.

Postoperative thyroid and cervical lymph node ultrasonography showed mildly enlarged lymph nodes in the left level IV region. A subsequent PET-CT scan was performed: (1) No abnormal density or hypermetabolic foci were observed in the postoperative areas of the left thyroid lobe and left lateral liver lobe. (2) Multiple nodules in both lungs showed no significant hypermetabolism; combined with the patient's history, these were considered highly suspicious for metastatic disease. Postoperative genetic testing revealed mutations in the NRAS, TP53, and TERT genes, with a low tumor mutational burden.

**Figure 3:**
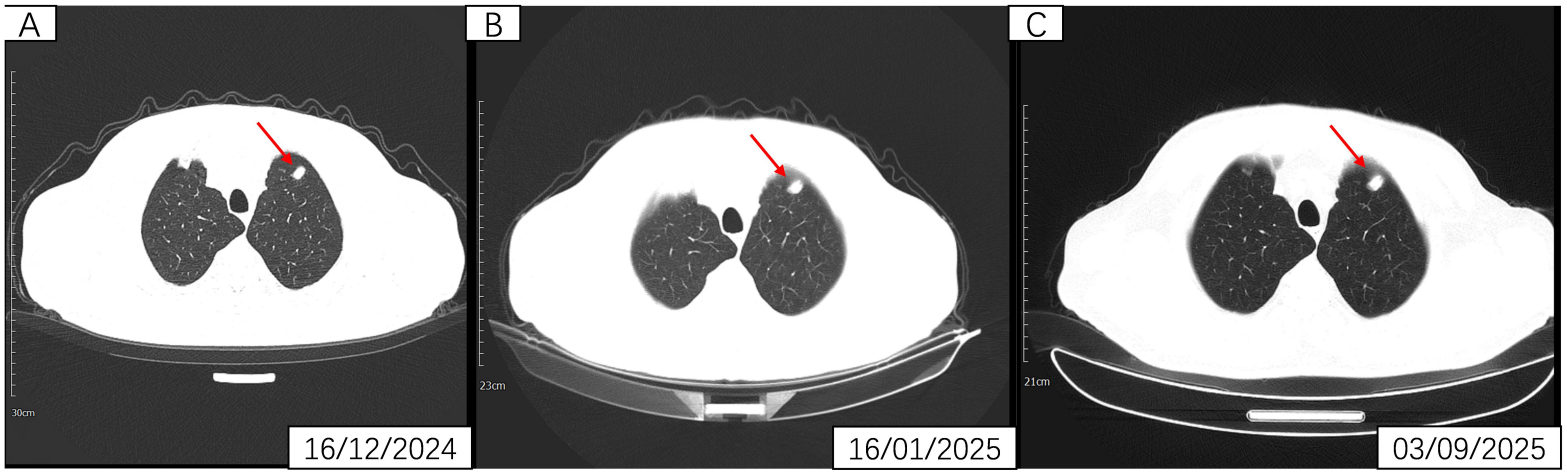
**(A-C)** Postoperative follow-up chest CT shows stable lung metastases with no significant change in size, taking a larger nodule as an example.

**Figure 4:**
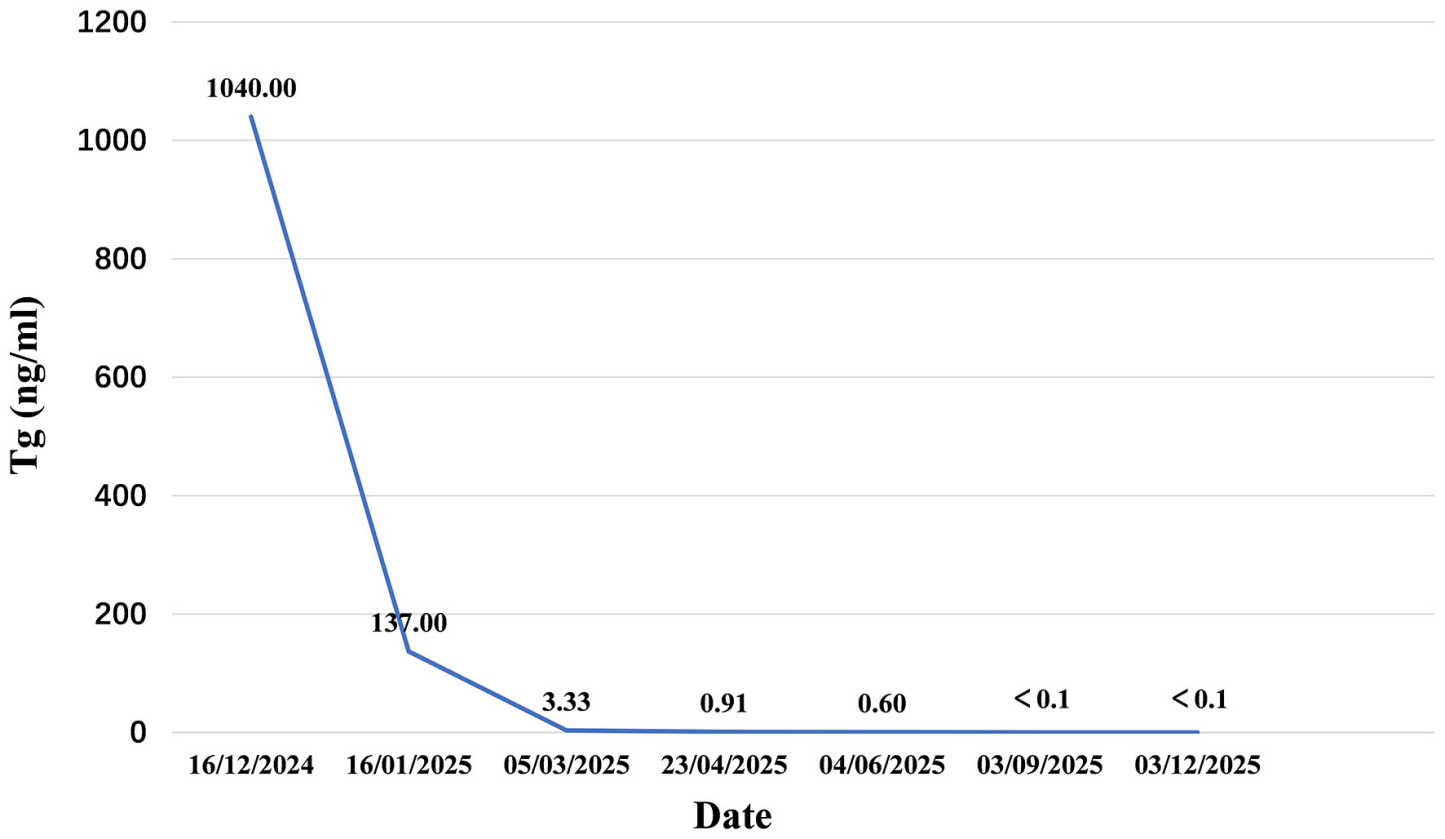
Under TSH suppression, an undetectable serum thyroglobulin level (< 0.2 ng/ml) combined with the absence of imaging abnormalities suggests possible complete remission.

**Figure 5:**
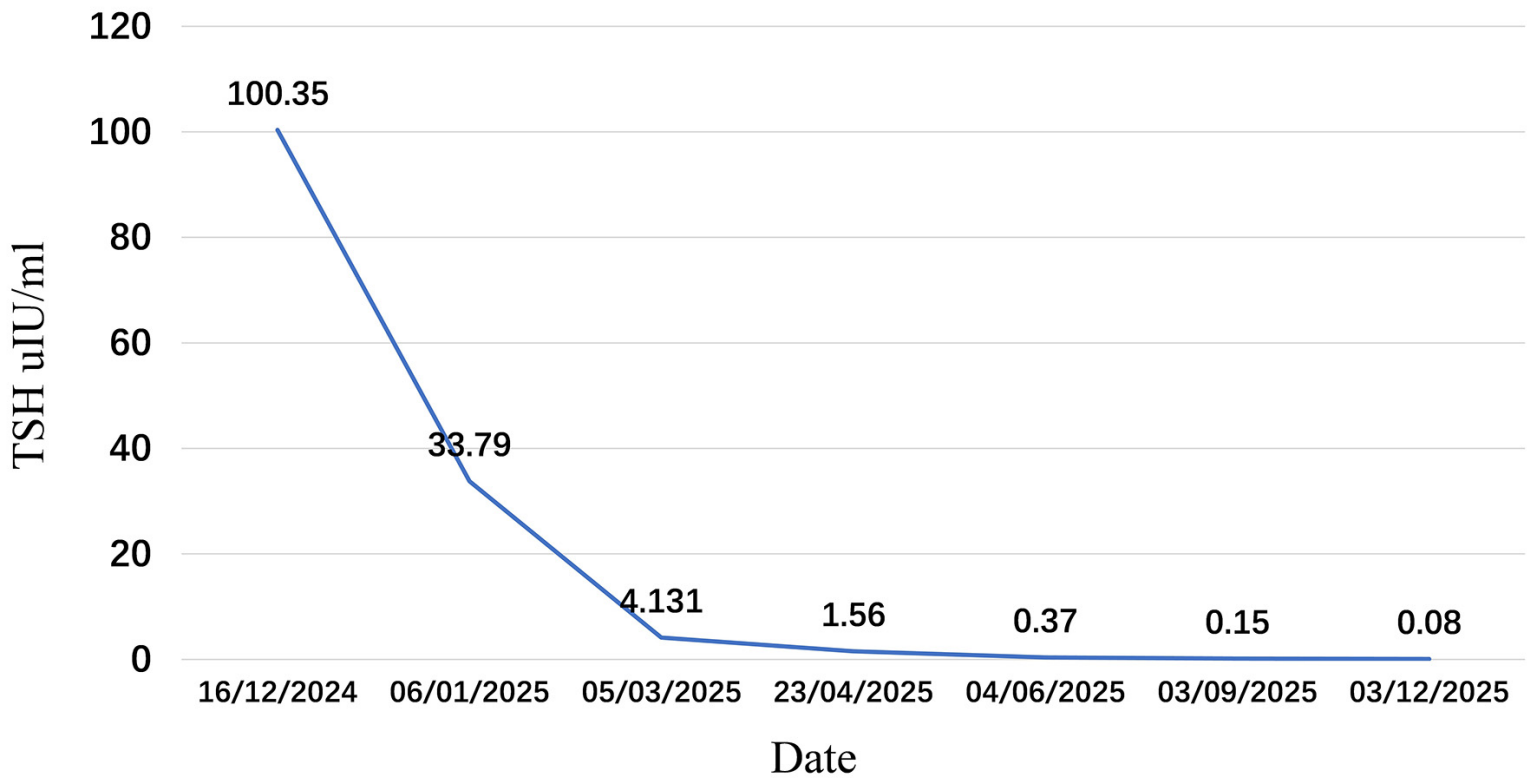
Following levothyroxine administration, serum TSH suppression was achieved to a target level of < 0.1 μIU/ml.

Following a second MDT discussion and evaluation, the following decisions were made: (1) Performance of completion thyroidectomy with lymph node dissection; subsequent postoperative pathology revealed no residual carcinoma. (2) To administer radioiodine therapy and initiate TSH suppression therapy after good postoperative recovery. (3) To recommend follow-up imaging for the lung nodules every 3–6 months. Follow-up to date has shown no significant progression (Figures 3–5).

### 2.2 Discussion

FTC is the second most common differentiated thyroid cancer, accounting for approximately 5%−10% (9) of all malignant thyroid tumors, with a peak incidence between 40 and 60 years of age and a female-to-male ratio of about 3:1. Histopathologically, the distinction between FTC and benign follicular adenoma relies entirely on the presence of capsular and/or vascular invasion. The overall prognosis of FTC is favorable. However, once distant metastasis occurs, the prognosis significantly worsens. Among different metastatic sites, the median overall survival (OS) for pulmonary metastasis is approximately 10 months, with a 5-year survival rate of about 21% (10). In contrast, hepatic metastasis is extremely rare, occurring in only 0.5–1% of FTC cases, and is associated with a poor prognosis (6). The rarity of hepatic metastasis, combined with extremely low survival rates, highlights severe clinical challenges posed by liver involvement. This highly aggressive clinical behavior is closely related to the underlying molecular driving mechanisms.

The pathogenesis of FTC follows a pattern of progressive accumulation of genetic alterations. The initiating event is typically a RAS gene point mutation or PAX8-PPARγ rearrangement, both of which converge on the MAPK signaling pathway, but alone are insufficient to confer full malignant potential (11). Transformation to invasive carcinoma requires the acquisition of additional late molecular events, most critically TERT promoter mutations and TP53 inactivation—the former mediates cellular immortalization through telomerase activation and is independently associated with distant metastasis and increased disease-specific mortality (12), while the latter eliminates p53-dependent tumor suppressor functions, promoting genomic instability. The metastatic lesion in this case harbored concurrent NRAS, TERT, and TP53 mutations: the NRAS mutation may have driven the initial formation of FT-UMP 8 years ago, while the superimposition of TERT and TP53 mutations conferred the ability for malignant transformation and hematogenous dissemination to the liver and lungs, thereby providing a mechanistic explanation for the discrepancy between the histologically “borderline” appearance of the primary lesion and its clinically aggressive behavior.

For FTC patients with distant metastasis involving multiple organs and complex clinical conditions, especially those with a history of multiple surgeries, an MDT should be established for comprehensive evaluation and decision-making. In this case, the first intervention of the MDT faced two core issues: clarifying the nature of the liver mass and pulmonary nodules, and formulating an individualized treatment plan.

#### 2.2.1 Regarding liver space-occupying lesions

Ligocka et al. (13) suggested that radical resection is the preferred treatment for liver oligometastases, which can significantly prolong recurrence-free survival. This finding has been confirmed by other reports (14, 15). In this case, the liver mass was located in the left lateral lobe, which meets the criteria for radical resection. To further clarify the nature of liver lesion, we chose robot-assisted surgical resection. While ensuring radical resection, this surgical method features precise anatomical dissection and relatively rapid postoperative recovery. It creates favorable conditions for subsequent treatment. Surgery was successfully completed, and postoperative pathology confirmed FTC metastasis. Timely review and confirmation were performed to avoid misdiagnosis. This pathological diagnosis prompted the MDT to conduct a critical reassessment of the original thyroid pathology from 8 years ago. This case illustrates that FTC shows a variety of histological characteristics, from well-differentiated follicular structure (similar to normal thyroid) to malignant characteristics. Due to their diverse subtypes, it may sometimes be challenging to distinguish between benign and malignant lesions by relying only on cytomorphology and tissue structure. Some scholars propose that the presence of metastasis indicates a biologically malignant tumor, even when both the primary and metastatic follicular adenomas show benign histology and a prolonged clinical course (16, 17). In this case, the postoperative pathology from the first thyroid surgery was cautiously interpreted as a follicular thyroid neoplasm of uncertain malignant potential. Based on the aforementioned perspective, we consider that the lesion 8 years ago should be regarded as FTC, given that the liver mass appearing 8 years later was confirmed to be a metastatic focus of FTC.

#### 2.2.2 Regarding pulmonary nodules

The MDT integrated the molecular profile into anatomical and functional assessments to formulate the following decisions: (1) not to perform a lung nodule biopsy, but instead to base the next steps on the postoperative pathological results of the liver lesion; (2) not to consider surgical resection initially, as the presence of multiple small lung nodules (the largest measuring 8 mm × 6 mm) precluded R0 resection regardless of molecular risk; and (3) following evaluation, to recommend ^131^I therapy as the main treatment after the liver mass was confirmed as metastatic FTC. Subsequent findings supported these decisions. Whole-body positron emission tomography-computed tomography suggested that the multiple lung nodules were metastatic foci, and the diagnosis of metastatic FTC was subsequently confirmed by postoperative pathology of the resected liver mass. Additionally, ^131^I whole-body scintigraphy can identify iodine-avid lesions, which is useful for assessing residual and recurrent disease after total thyroidectomy and the iodine avidity of distant metastases. Based on this characteristic, SPECT/CT revealed that most of the lung nodules were iodine-avid. Thus, iodine avidity and low TMB supported RAI as optimal therapy, while TERT/TP53 mutations, without overriding unresectability, mandated closer surveillance (every 3–6 months). Furthermore, there was no significant change in the size of pulmonary metastases within 12 months after treatment (Figure 3). Published evidence supports this approach. Moneke et al. (18) reported that surgical resection is recommended for isolated, solitary distant metastatic lesions with a relatively high safety profile. However, in this case, the presence of multiple small lung nodules precluded R0 resection, and surgical removal was therefore not considered initially. This treatment has demonstrated significant efficacy for lung metastasis in FTC with distant dissemination, with some patients achieving complete pathological remission. Given iodine avidity, the treatment response for micrometastases is generally better than for larger nodular lesions (19). Thus, for the lung nodules in this case, radioiodine therapy was considered superior to surgical intervention. Notably, this stable disease course following radioiodine therapy, despite the absence of significant radiographic regression, is consistent with the recognized pattern of prolonged indolent progression in iodine-avid pulmonary micrometastases from FTC, where disease stabilization rather than rapid clearance often translates into favorable long-term outcomes, as the 10-year overall survival for patients achieving complete metabolic response or stable disease with radioactive iodine (RAI) exceeds 90% (20).

#### 2.2.3 Regarding comprehensive treatment

Under the coordination of the MDT, the hepatic and pulmonary metastases were controlled through surgical resection and radioactive iodine therapy, respectively. On this basis, TSH suppression therapy was incorporated into the comprehensive treatment plan as the endocrine cornerstone of long-term postoperative FTC management. Studies have shown that the combination of ^131^I treatment with TSH suppression treatment can significantly prolong the disease-free survival of patients (21). TSH promotes thyroid cell growth, including tumor cells. Studies indicate that the activation of this process enhances cellular proliferation by increasing the expression of thyroid-related proteins, including Tg (22). There is a broad consensus in the scientific community that elevated TSH levels have a role in promoting thyroid cell proliferation, with the potential to contribute to distant metastasis (23, 24). Therefore, to prevent the progression of FTC, TSH suppression therapy was selected as part of the comprehensive treatment plan for this case. This patient was classified as high-risk group for tumor recurrence, and it was recommended to maintain TSH levels below 0.1 μIU/ml (25). Gubbi et al. (26) observed that, while TSH suppression therapy may not enhance the overall survival of high-risk patients, it can markedly reduce the risk of recurrence. During follow-up, the risk of tumor recurrence may change for some patients as treatment progresses. Therefore, the target for TSH suppression should be adjusted based on dynamic monitoring of Tg levels, imaging examinations, and other factors. Accordingly, in this case, postoperative Tg and TSH levels were maintained within optimal ranges (Figures 4, 5), and no evidence of recurrence was observed on imaging within 12 months.

Although TSH suppression and radioiodine therapy have achieved a favorable initial response, the high-risk molecular profile with concurrent TERT promoter and TP53 mutations warrants consideration of future targeted therapy (12). Multi-targeted tyrosine kinase inhibitors (TKIs), such as lenvatinib and sorafenib, are the recommended first-line systemic therapy for progressive, radioiodine-refractory differentiated thyroid cancer (RAIR DTC), with lenvatinib demonstrating a median overall survival of 38.9 months in real-world studies (27). The 2025 ATA guidelines operationalize this decision through the DATA framework's Assessment of response (A), which classifies outcomes into four categories: excellent response (suppressed Tg < 0.2 ng/ml with negative imaging), indeterminate response, biochemical incomplete response, and structural incomplete response (8). Current guidelines recommend initiating TKI therapy only when patients develop symptomatic or structurally progressive disease (28). In this case, at 12 months after RAI therapy, the patient demonstrated suppressed Tg < 0.1 ng/ml (Figure 4), stable pulmonary nodules on CT (Figure 3), and iodine avidity on SPECT/CT, meeting criteria for an excellent response. The 2025 guidelines explicitly reserve TKI for structural or biochemical incomplete response, while patients achieving excellent response should continue TSH suppression and close surveillance. In this case, targeted therapy was not initiated. It is important to emphasize that the presence of TERT promoter and TP53 mutations did not indicate RAI refractoriness or mandate immediate TKI therapy; rather, these alterations served as prognostic stratifiers that intensified surveillance rather than altered the first-line therapeutic modality. The patient's pulmonary metastases demonstrated iodine avidity on post-therapy scintigraphy, and follow-up chest CT over 12 months showed no significant progression. These findings are consistent with ongoing RAI responsiveness rather than RAI refractoriness; therefore, the clinical threshold for initiating TKI therapy was not reached.

### 2.3 Conclusion

This case describes the progression of a follicular tumor of uncertain malignant potential (FT-UMP) to widely metastatic FTC, presenting with synchronous hepatic and pulmonary metastases 8 years after thyroidectomy. The concurrent identification of NRAS, TERT, and TP53 mutations in the metastatic lesion provides a molecular rationale for this aggressive phenotype, challenging the conventional assumption that FT-UMP follows an invariably indolent course. Three clinical implications follow. First, patients with FT-UMP require prolonged postoperative surveillance, including serial thyroglobulin measurement and radiological imaging, even when initial histopathology shows no definitive capsular or vascular invasion. Second, comprehensive genomic profiling may improve risk stratification in borderline follicular neoplasms; the detection of TERT promoter or TP53 alterations should prompt intensified surveillance, regardless of histological evidence of malignancy. Third, MDT involvement should be maintained throughout the disease continuum, from initial diagnostic evaluation of metastatic disease to individualized therapeutic planning and long-term follow-up. The 12-month follow-up demonstrates initial treatment response but remains insufficient to assess long-term prognosis or delayed progression; sustained surveillance is warranted.

## Data Availability

The original contributions presented in the study are included in the article/supplementary material, further inquiries can be directed to the corresponding author.
